# Constructing a Knowledge-Based Database for Dermatological Integrative Medical Information

**DOI:** 10.1155/2013/649040

**Published:** 2013-12-09

**Authors:** Jeeyoung Shin, Yunju Jo, Hyunsu Bae, Moochang Hong, Minkyu Shin, Yangseok Kim

**Affiliations:** Department of Physiology, College of Korean Medicine, Kyung Hee University, Seoul 130-701, Republic of Korea

## Abstract

Recently, overuse of steroids and immunosuppressive drugs has produced incurable dermatological health problems. Traditional medical approaches have been studied for alternative solutions. However, accessing relevant information is difficult given the differences in information for western medicine (WM) and traditional medicine (TM). Therefore, an integrated medical information infrastructure must be utilized to bridge western and traditional treatments. In this study, WM and TM information was collected based on literature searches and information from internet databases on dermatological issues. Additionally, definitions for unified terminology and disease categorization based on individual cases were generated. Also a searchable database system was established that may be a possible model system for integrating both WM and TM medical information on dermatological conditions. Such a system will yield benefits for researchers and facilitate the best possible medical solutions for patients. The DIMI is freely available online.

## 1. Introduction 

It has recently been shown that overuse of steroids and immunosuppressive drugs as well as conventional treatment may cause chronic and incurable dermatological illnesses, which raises the issues of side effects and tolerance from excessive use. Further, unverified information for treating chronic illnesses wastes patients' money and time. In addition, it seriously affects their quality of life and requires long-term dedicated care with immense socioeconomic cost [1, 2].

Whereas traditional medicine (TM) focuses on tracking and curing the root of dermatological conditions caused by internal organ malfunction, western medicine (WM) focuses on treating symptoms, which relies on treatments that produce acute and immediate results through antibiotics and anti-inflammatory medicine. Consequently, if patients only rely on antibiotics, the skin's own immune system can be oppressed and subdued, which leads to its malfunction and eventual development of chronic, hard-to-cure dermatological conditions. Thus, overusing antibiotics for a long period of time can decrease skin's immune function and lead to antibiotic tolerance, side effects, and dyspepsia [3]. Therefore, integrative medicine has been recommended as an alternative method that combines WM, TM, and complementary and alternative medicine (CAM).

In general, TM stresses the importance of catering therapy to each individual's needs, as opposed to western therapeutic approaches that are standardized and stress “average” efficacy in large, double-blind, placebo-controlled studies [4]. Many WM and TM areas provide opportunities for productive and collaborative research. In designing such trials in the future, it is important to incorporate concepts from both WM disease identification and pathogenesis as well as TM syndrome identification and management into research protocols. Such integration will require collaboration between WM and TM practitioners, sharing the development of protocols, treatment, and information exchange as well as strengthening interpersonal connectivity and leadership [5]. Therefore, it is necessary to construct an integrated medical infrastructure to utilize and facilitate integration between western and tradition treatments.

The Diseases Database, Medline Plus, and eMedicine Medscape are representative western medical professional databases, which grant universal information access for entire diseases rather than limited information [6–8]. Information on TM only includes ancient document databases, herbal databases, and traditional medicine-related references, which can be limited for applying and utilizing such data. Data mining and TM and WM database integration are important but problematic steps. Problems include heterogeneity in the data format and structures as well as no standard terminology. Cultural and linguistic differences further complicate data integration [9]. Moreover, TM disease names for dermatological conditions can be vast and vague; thus, the terminology must be systematically categorized based on the underlying concepts. Standardization through comparing the relative terminology for dermatology conditions in Korean, Chinese, and English is necessary. Thus, to more easily access dermatology-related integrated information, it is necessary to construct a database and information system with character string and keyword search capabilities.

The goal for such studies is to treat patients and illnesses by combining TM and WM techniques for the two disciplines to complement each other. This study will use such commonly accepted notions and propose a model for the future [5]. Therefore, this research was devised to construct an integrated information system for dermatological conditions and provide easier access to and facilitate related research processes, which may enable researchers to generate better alternative treatments based on WM and TM.

## 2. Methods 

### 2.1. Data Source Extraction

For this study, WM and TM information was collected based on bibliographic literature and internet database searches related to dermatological issues as one source for constructing a knowledge-based database.

First, the WM literature search references are from “Dermatology”, “Fitzpatrick's dermatology in general medicine” and “Andrews' diseases of the skin: clinical dermatology” [10–12]. The WM internet database information was excerpted from PubMed, the Diseases Database, Medline Plus, and eMedicine Medscape. WM information, such as definitions, causes, classifications, symptoms, diagnoses, and treatments, was retrieved from the aforementioned references and internet databases.

Second, a TM literature search for related dermatological references was excerpted from “Dermatology of Korean medicine,” “Text of traditional Korean dermatology,” “Dermatology and venereal disease of traditional Chinese medicine,” “Dermatology of TCM,” “Collection of clinical experience of Zhao Bingnan,” “Collection of clinical experience of Zhou Renkang,” and “Collection of clinical experience of Zhang Zhili” [13–19]. The TM internet database was based on Pharmacopoeia and Natural Drug Standards of Korea, a natural herbs database system provided by MFDS and the Korean Traditional Knowledge Portal, which provide prescriptions and herb-related information [20, 21]. TM information, such as syndromes, prescriptions, and composition differentiation, was extracted from the aforementioned references and internet databases.

### 2.2. Define Unified Terminology

TM diagnoses individual diseases using the “syndrome identification” process. The theory underlying TM diagnosis and management has not been clearly articulated in western scientific terms, but the TM syndrome identification process is believed valid by its practitioners [5]. Disease names for TM dermatology conditions can be vast and vague; thus, systematic terminology categorization is necessary. Standardization by comparing relative terminology for dermatology conditions in Korean, Chinese, and English is necessary.

Medical Subjects Headings (MeSH) was used, which categorizes medical terminology in Medline. One representative term from each disease name was selected as the “primary key” for synonym searches; related information can be searched using the primary key. For example, “dyshidrotic eczema” can be searched using “acute vesiculobullous hand eczema,” “cheiropompholyx”, “podopompholyx,” and “pompholyx”. Thus, if “pompholyx” is input, it is transformed into “dyshidrotic eczema,” and the related information can be searched.

On the other hand, a number of studies have shown that TM is useful in treating skin diseases [4]. For example, WM diseases, such as endocrine disorder, are unlikely to be matched with related diseases. Therefore, because dermatological terminology from TM and WM is not always the same, a matching process using similar symptoms was implemented. Thus, a search for one TM disease is likely to have more than one corresponding WM disease name.

Considering such conditions, entire disease names have been categorized under 5 subdivisions: TM disease names (Korean), TM disease names (Chinese), WM disease names (Korean), WM disease names (English), and WM disease synonyms (English). The database was designed to yield the same results from searches using any of the five subdivisions.

### 2.3. Categorizing

For the Korean Classification of Diseases (KCD) code, a revised version of the 10th revision of the International Statistical Classification of Diseases and Related Health Problems (ICD-10) for Korean uses was adopted as the national standard for KCD codes in hospitals. This code system has been used for KCD statistics in various nations and insurance claims. However, ICD-10 was originally developed to categorize diseases and causes, not to subdivide KCD. Consequently, KCD categorization is not sophisticated enough for clinical purposes. Therefore, in our DIMI database, 24 categories were constructed based on causes for various dermatological conditions and symptoms, which were codified using letters from A to X. The database includes 135 TM and 153 WM dermatological conditions in their corresponding categories.

### 2.4. Database Construction & the Information Search Process

#### 2.4.1. Logically Structuring the Database

This system fully consolidated traditional and western medicinal information utilizing a database and disease names as the primary keys, which are critical for integrated data. Further, it includes a logical structure to facilitate prescription searches as well as information related to ordinary diseases.

#### 2.4.2. Setting Up Database

The system includes every type of disease for single diseases indicated by various titles. A verification process was used, which verified that the information in the database was correctly input, and comparison processes for the original text data were used. This process also verified the accuracy of the uploaded information; a comparison of the full, original text was used.

#### 2.4.3. Structuring the Interface and Developing the Interface for Database Searching

It is recommended that illustrative explanations and detailed information on the processed data are included in the database, which will reduce the user confusion. A basic search interface utilizing disease and prescription names is provided. A search function was constructed that allows users to search information based on various queries, such as using traditional (Korean and Chinese searches) and western (Korean and English searches) terminology, WM disease synonyms (English searches), and prescriptions (Korean and Chinese searches). The search functions were designed based on the need to identify various queries for disease and prescription names.

**Figure 1:**
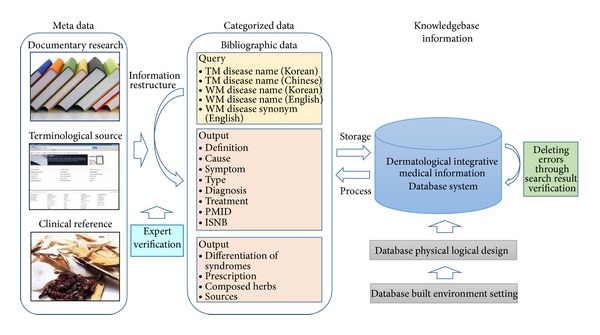
The DIMI database system flowchart.

To build the search interface, the Application Service Provider (ASP) was necessary, and to build and maintain the database, Microsoft SQL server 2008 was used. The flowchart for Dermatological Integrative Medical Information (DIMI) Database System is shown in Figure 1.

## 3. Results

DIMI is the WM-TM combined knowledge-based database established to consolidate and integrate dermatology-related information, facilitate research processes, and indicate possible alternative treatments. The system includes a clearer and more defined information system by categorizing western-traditional medical terminology. Further, an easy-to-access information system was constructed through a systematic, integrated information database.

Categorizing western-traditional dermatology information into 24 subdivisions based on condition causes, 153 WM and 135 TM diseases were matched according to their symptoms.

Western definitions, causes, classifications, symptoms, diagnoses, and treatments as well as traditional syndromes and 1048 prescriptions as well as composed herbs were stored in the database. Further, the references are hyperlinked to the PMID and ISBN; thus, a researcher can find PubMed searches, which allows users to find references for the latest health professional articles on a topic.

**Figure 2:**
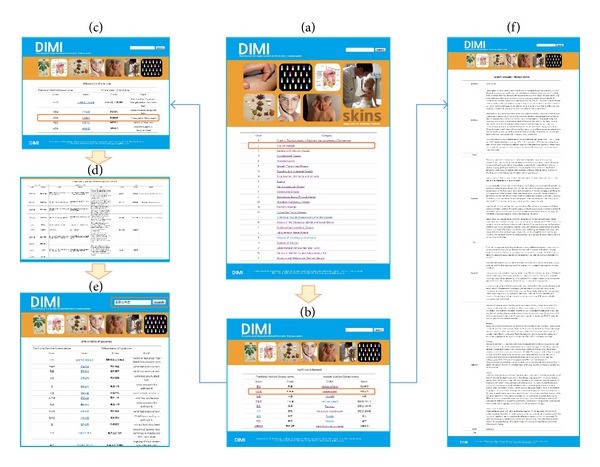
The primary pages in DIMI. (a) The screenshot for the main page that features 24 categorized subdivisions. (b) The screenshot for one of 24 subdivisions that has related disease names. (c) The screenshot that shows TM differentiations of syndromes when TM disease names are clicked. (d) The screenshot that shows prescriptions, composed herbs, and sources when TM differentiations of syndromes are clicked. (e) The screenshot that shows every related TM disease name and differentiation of syndromes when searched with prescription names. (f) The screenshot that shows WM synonyms, definition, cause, symptom, type, diagnosis, treatment, PMID, and ISBN when WM disease names are clicked.

This database includes links to related information. For disease name searches, related WM-TM integrated information can be retrieved. For prescription name searches, various applicable diseases using a specific prescription can be retrieved as an integrated source for users (Figure 2).

## 4. Discussion 

Due to the digital innovation in the Internet and its applications, the field of medicine has been evolved to meet each patient's needs retrieving their past medical records. Currently, the same amount and type of medication are administered to patients if they are diagnosed with the same illness. Now, that is to be changed since each patient's case can be treated individually utilizing their biodata stored in the computing system. The digitization of patients' medical records can improve the quality of medical service. The accumulated medical data, of course, must be converted into sources that can be immediately used in medical practice. In order to achieve that goal, it is important to establish systematized knowledge-based search systems. This study has been designed to be primary sources of information for researchers to find alternative treatments and conduct relevant researches.

The main focus of the study is to match the names of illnesses in TM with equivalent names in WM, accumulating a vast amount of relevant information comparing symptoms of individual illnesses, based on literature researches and web database searches on the subject of dermatological problems. The causes of illnesses and their symptoms have been categorized and codified with alphabet letters. Also all traditional medical problems and their western equivalents have been included under respective categories. So the information in WM can be retrieved based on the definitions, causes, classifications, symptoms, diagnoses, treatments and references. Plus, the information in TM has been organized based on TM differentiation of syndrome and prescribed medications. To build a searchable interface, Application Service Provider (ASP) was used, and to build and maintain a database, Microsoft SQL Server 2008 was used.

The information in dermatological conditions has been categorized into 24 subdivisions. 135 names of illnesses in TM and 153 equivalent names in WM have been matched, based on symptoms. The data have been categorized again based on their TM disease names (Korean), TM disease names (Chinese), WM disease names (Korean), WM disease names (English), and WM disease synonyms (English) to clarify and accelerate the data processing. The database of TM consists of 1048 prescriptions and the information on composed herbs, utilizing TM differentiation of syndrome. In particular, among those registered prescriptions, Wuwei Xiaodu Yin (Antiphlogistic Decoction of Five Drugs), Xiaofeng San (Powder for Dispersing Pathogenic Wind), Huanglian Jiedu Tang (Antidotal Decoction of Coptis), Longdan Xiegan Tang (Decoction of Getiana for Purging), and Xijiao Dihuang Tang (Decoction of Rhinoceros Horn and Rehmannia) top the list raising hopes on their medical applications in dermatological studies. The database of WM consists of definitions, causes, classifications, symptoms, diagnoses, and treatments. If references are clicked linking PMID, PubMed Page is directly connected, which means that every information is systematically interconnected. If web searches are conducted via TM prescription names, the names of illnesses with those prescriptions can be retrieved so users are provided with integrated information.

Currently, the main focus of TM-related database is limited within classic literature DB and searches for dissertations in Korean medicine. However, the applications of the current database are limited. Also, they only record string. If string inserted by users is not matched with the database, relevant information may not be retrieved. Inevitably, systematically integrated web search systems should be established based on categorized knowledge bases so that not only medical professions but also nonprofessionals can easily access TM information. In terms of treatments, many folk prescriptions and TM are effective to treat certain rare or severe disease. And collecting those formulae or medicine will be an urgent task. The studies on TCM database fields including TCMID [22], HIT [23], TCM@Taiwan [24], and TCMGeneDIT [25] which contain prescriptions, herbs, compounds, drugs, diseases, and related targets only show superficially analyzed pharmaceutical information.

Thus, the establishment of this sort of database, the integration of both TM and WM clinical information providing DIMI, has never been tried before. The focus of the system is also on providing researchers with options they can choose to find clinical information they can actually use in their field medical practice. The data are based on already-verified research results and ruling out false positive. So researchers can save time and patients and their family members can access right medical information. The data can also be applied in many different fields of medicine maximizing their potentials. Currently, the information can be retrieved only through the Internet. It should be further studied to expand the possible usages via e-books and mobile devices as well. Also, the database should be regularly updated and expanded with new research and study results to make them up-to-date.

## 5. Conclusion

A searchable database system was established as a model system for integrating both TM and WM information on dermatological conditions, which takes advantage of both TM and WM to provide the best possible medical solutions and patient treatments. This research will benefit both WM and TM dermatology research, which will facilitate development of new treatments. DIMI is freely available on http://www.dimi.or.kr/.

## References

[1] Li FL, Wang YF, Li X (2012). Characteristics and clinical managements of chronic skin ulcers based on traditional chinese medicine. *Evidence-Based Complementary and Alternative Medicine*.

[2] Tang MM, Chang CC, Chan LC, Heng A (2013). Quality of life and cost of illness in patients with psoriasis in Malaysia: a multicenter study. *International Journal of Dermatology*.

[3] Fung AYP, Look PCN, Chong L-Y, But PPH, Wong E (1999). A controlled trial of traditional Chinese herbal medicine in Chinese patients with recalcitrant atopic dermatitis. *International Journal of Dermatology*.

[4] Koo J, Desai R (2003). Traditional Chinese medicine in dermatology. *Dermatologic Therapy*.

[5] Chen K, Xu H (2003). The integration of traditional Chinese medicine and Western medicine. *European Review*.

[6] Diseases Database. http://www.diseasesdatabase.com/content/.

[7] Medline Plus. http://www.nlm.nih.gov/medlineplus/skinconditions.html/.

[8] eMedicine Medscape. http://emedicine.medscape.com/.

[9] Cheung KH, Chen H (2010). Semantic Internet for data harmonization in Chinese medicine. *Chinese Medicine*.

[10] Rapini RP, Bolognia JL, Jorizzo JL (2007). *Dermatology: 2-Volume Set*.

[11] Freedberg IM, Goldsmith LA, Katz S, Austen KF, Wolff K (2003). *Fitzpatrick's Dermatology in General Medicine*.

[12] James W, Berger T, Elston D (2005). *Andrews' Diseases of the Skin: Clinical Dermatology*.

[13] Korean Dermatological Association *Dermatology*.

[14] Ko WS *Text of Traditional Korean Dermatology and Surgery*.

[15] Yu WQ, Tan YJ, Hwang TK *Dermatology and Venereal Disease of Traditional Chinese Medicine*.

[16] Zhao XH *Dermatology of TCM*.

[17] Beijing Hospital of TCM *Collection of Clinical Experience of Zhao Bingnan*.

[18] Guang'anmen Hospital of China Academy of Chinese Medical Sciences *Collection of Clinical Experience of Zhou Renkang*.

[19] Zhang ZL *Collection of Clinical Experience of Zhang Zhili*.

[20] Ministry of Food and Drug Safety. http://www.mfds.go.kr/herbmed/index.do.

[21] Korean Traditional Knowledge Portal. http://www.koreantk.com/.

[22] Xue RC, Fang Z, Zhang MX, Yi ZH, Wen CP, Shi TL (2013). TCMID: traditional Chinese medicine integrative database for herb molecular mechanism analysis. *Nucleic Acids Research*.

[23] Ye H, Ye L, Kang H (2011). HIT: linking herbal active ingredients to targets. *Nucleic Acids Research*.

[24] Chen CY-C (2011). TCM Database@Taiwan: the world’s largest traditional Chinese medicine database for drug screening in silico. *PLoS ONE*.

[25] Fang Y-C, Huang H-C, Chen H-H, Juan H-F (2008). TCMGeneDIT: a database for associated traditional Chinese medicine, gene and disease information using text mining. *BMC Complementary and Alternative Medicine*.

